# Supplementary material for: Functional Characterization of Transforming Growth Factor-β Signaling in Dasatinib Resistance and Pre-BCR+ Acute Lymphoblastic Leukemia
Source: Cancers (Basel). 2023 Aug 30;15(17):4328. doi: 10.3390/cancers15174328 (PMC10486903; doi:10.3390/cancers15174328)
Supplement: Supplementary file 1 [file cancers-15-04328-s001.zip › cancers-2531534-Supplementary Materials.pdf]

Supplementary Figures

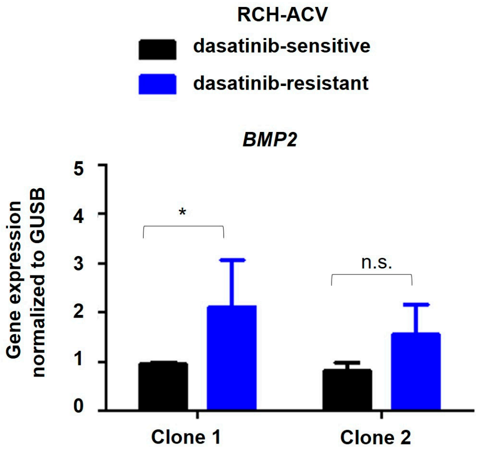

Figure S1. Gene expression of TGFβ pathway member *BMP2* was validated using RT-qPCR. *GUSB* was used as housekeeping gene. Expression of target gene is displayed as normalized to housekeeping gene mean concentration. Data is shown as mean of three replicate experiments with error bars indicating ± SD. Statistical analysis by Student's *t*-test (*, p < 0.05; ***, p < 0.001).

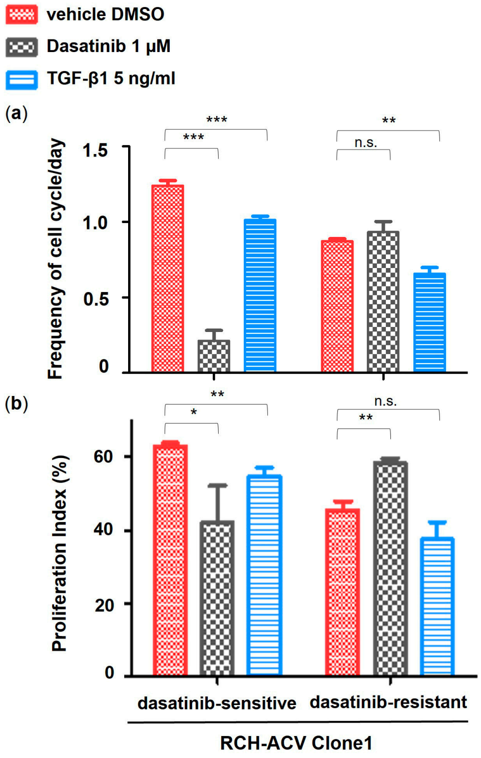

Figure S2. Dasatinib–sensitive RCH-ACV cells compared to resistant cells show a reduced frequency of cell cycles per day in the presence of dasatinib. Comparative pharmacological long-term treatment in dasatinib-sensitive and –resistant RCH-ACV cell line after 6 days of treatment with vehicle, dasatinib and TGF-β1. (**a**) Bar graphs represent the frequency of cell cycles per day after 6 days of treatment with the indicated concentrations. (**b**) Bar graphs represent the proliferation index at day 6 quantified by cell cycle flow cytometry analysis. Bars represent the mean of three independent experiments with error bars indicating ± SD. Statistical analysis by Student's *t*-test (*, p < 0.05; **, p < 0.01; ***, p < 0.001; n.s., not significant).

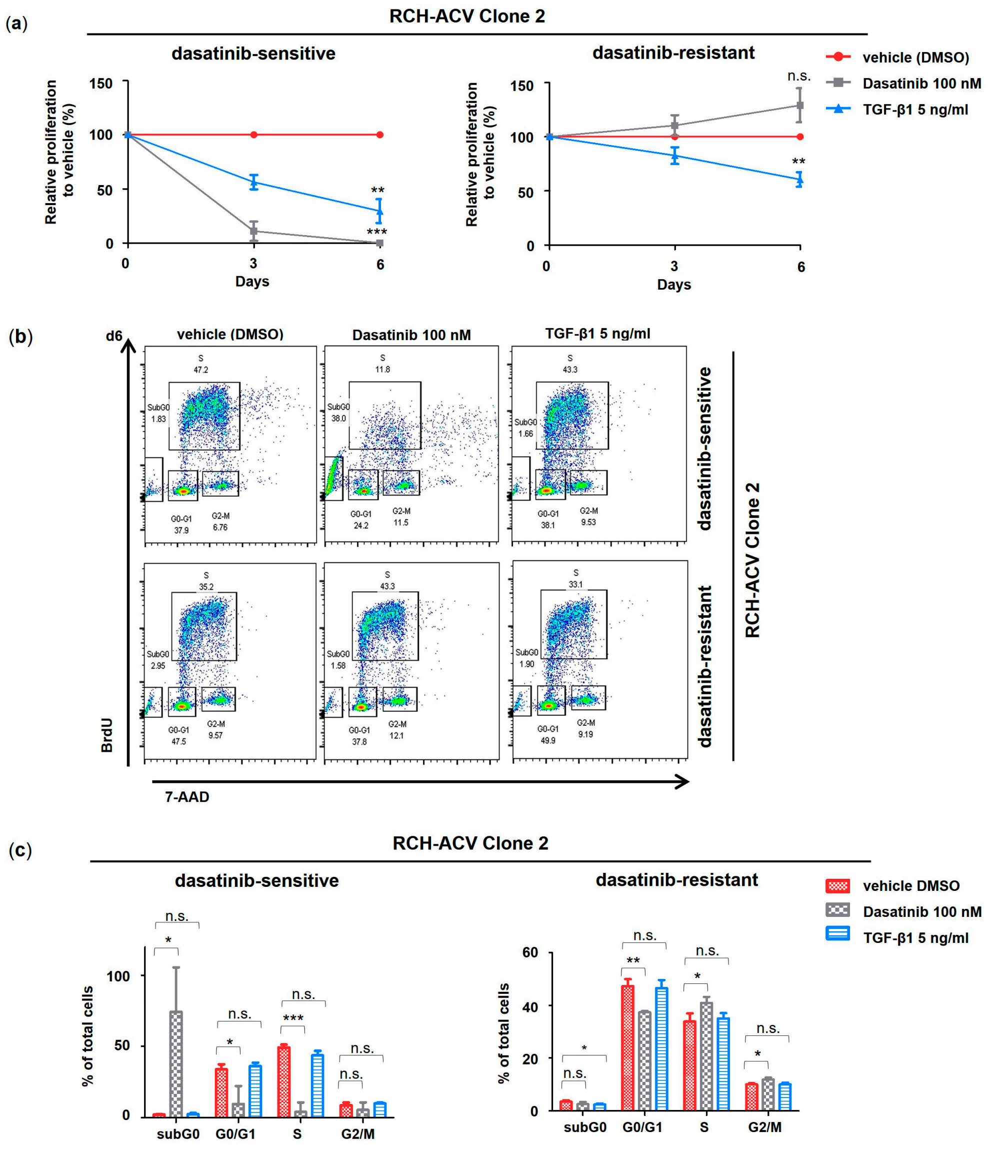

Figure S3. TGF-β1 inhibits cell proliferation in E2A-PBX1+ dasatinib–sensitive and -resistant RCH-ACV cells. Comparative pharmacological long-term treatment in E2A-PBX1+ dasatinib-sensitive and –resistant RCH-ACV clone 2 over 6 days with vehicle, dasatinib and TGF-β1. The concentration of TGF-β1 was optimized to detect an effect in cell cycle and apoptosis analysis. Cell proliferation was markedly reduced at concentrations of 0.5 and 1.0 ng/ml, however, correlating changes in cell cycle or apoptosis assays were detected at 5.0 and 10.0 ng/ml by flow cytometry. Further experiments were performed with TGF-β1 5.0 ng/ml. (**a**) Cell proliferation is expressed as relative to vehicle treated cells calculated from accumulative data. (**b**) Flow cytometry plots of cell cycle analysis at day 6 representative of three independent experiments. BrdU, bromodeoxyuridine; 7-AAD, 7-aminoactinomycin D. (**c**) Bar graphs represent quantification of cell cycle analysis at day 6. Data is shown as mean of three independent experiments with error bars indicating ± SD. Statistical analysis by Student's *t*-test (*, $p < 0.05$; **, $p < 0.01$; ***, $p < 0.001$; n.s., not significant).

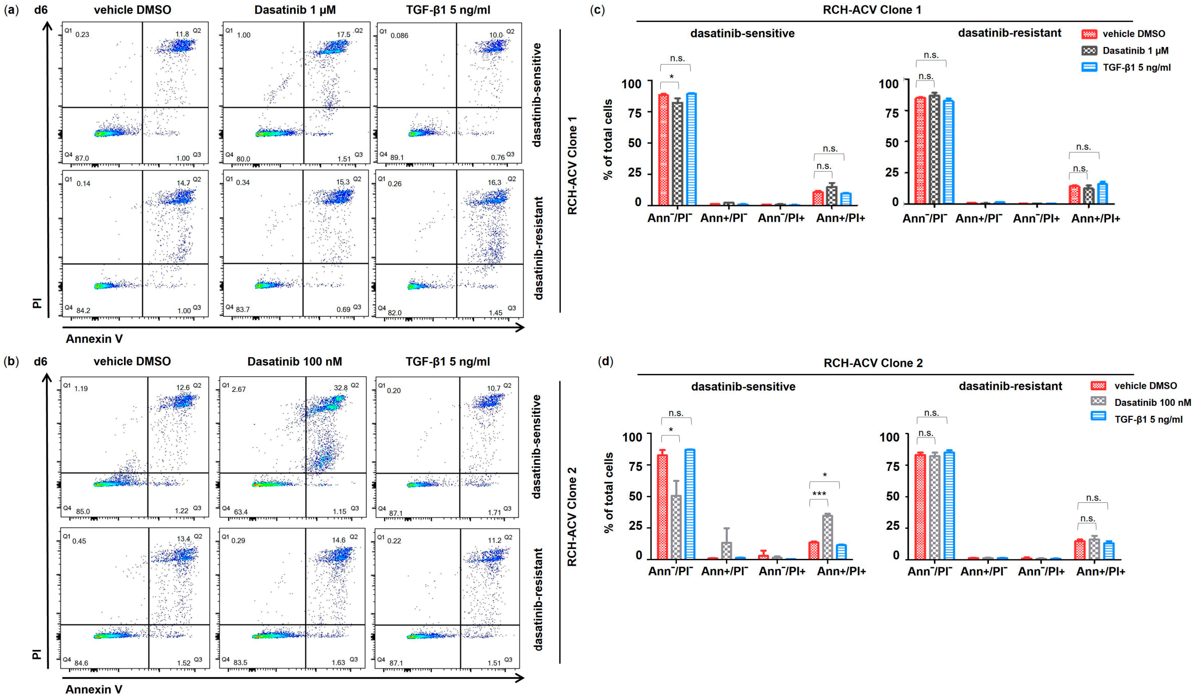

Figure S4. Dasatinib, but not TGF-β1, induces cell death in dasatinib-sensitive RCH-ACV cells. AnnexinV/PI Apoptosis assay in dasatinib-sensitive and –resistant RCH-ACV cells after 6 days of treatment with vehicle, dasatinib and TGF-β1. The concentration of TGF-β1 was optimized to detect an effect in cell cycle and apoptosis analysis. Cell proliferation was markedly reduced at concentrations of 0.5 and 1.0 ng/ml, however, correlating changes in cell cycle or apoptosis assays were detected at 5.0 and 10.0 ng/ml by flow cytometry. Further experiments were performed with TGF-β1 5.0 ng/ml. (**a**) **and** (**b**) Representative flow cytometry plots of apoptosis analysis of three independent experiments. PI, propidium iodide. (**c**) **and** (**d**) Bar graphs represent quantification of apoptosis analysis. Data is shown as mean of three independent experiments with error bars indicating ± SD. Statistical analysis by Student's *t*-test (*, $p < 0.05$; ***, $p < 0.001$; n.s., not significant). Ann, Annexin V; PI, propidium iodide.

Table S1. Primer pairs used in gene expression studies.

Gene target		Primer Sequence 5' -> 3'
GAPDH	F	GAGTCAACGGATTTGGTCGTATT
GAPDH	R	GAATTTGCCATGGGTGGAAT
GUSB	F	CGCCCTGCCTATCTGTATTC
GUSB	R	TCCCCACAGGGAGTGTGTAG
SMAD3	F	CCGATGTCCCCAGCACATAAT
SMAD3	R	CGCTGGTTCAGCTCGTAGTA
BMP2	F	CGCTCTTTCAATGGACGTGT
BMP2	R	AGCAGCAACGCTAGAAGACAG
c-MYC	F	CACCACCAGCAGCGACTCT
c-MYC	R	ACAGAAACAACATCGATTTCTTCCT
p15Ink4b	F	GGGAGGGTAATGAAGCTGAG
p15Ink4b	R	GGCCGTAAACTTAACGACACT
p21Cip1	F	TGGAGACTCTCAGGGTCGAAA
p21Cip1	R	GGCGTTTGGAGTGGTAGAAATC
