# Supplementary figures and images for: Functional Characterization of Transforming Growth Factor-β Signaling in Dasatinib Resistance and Pre-BCR+ Acute Lymphoblastic Leukemia
Source: Cancers (Basel). 2023 Aug 30;15(17):4328. doi: 10.3390/cancers15174328 (PMC10486903; doi:10.3390/cancers15174328)

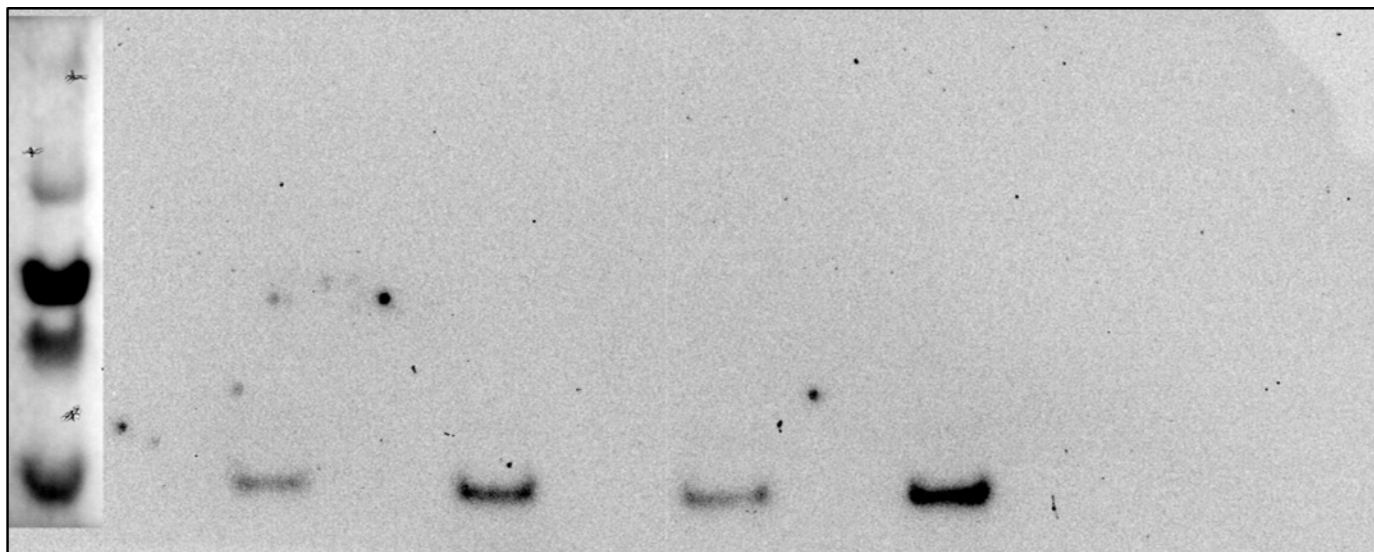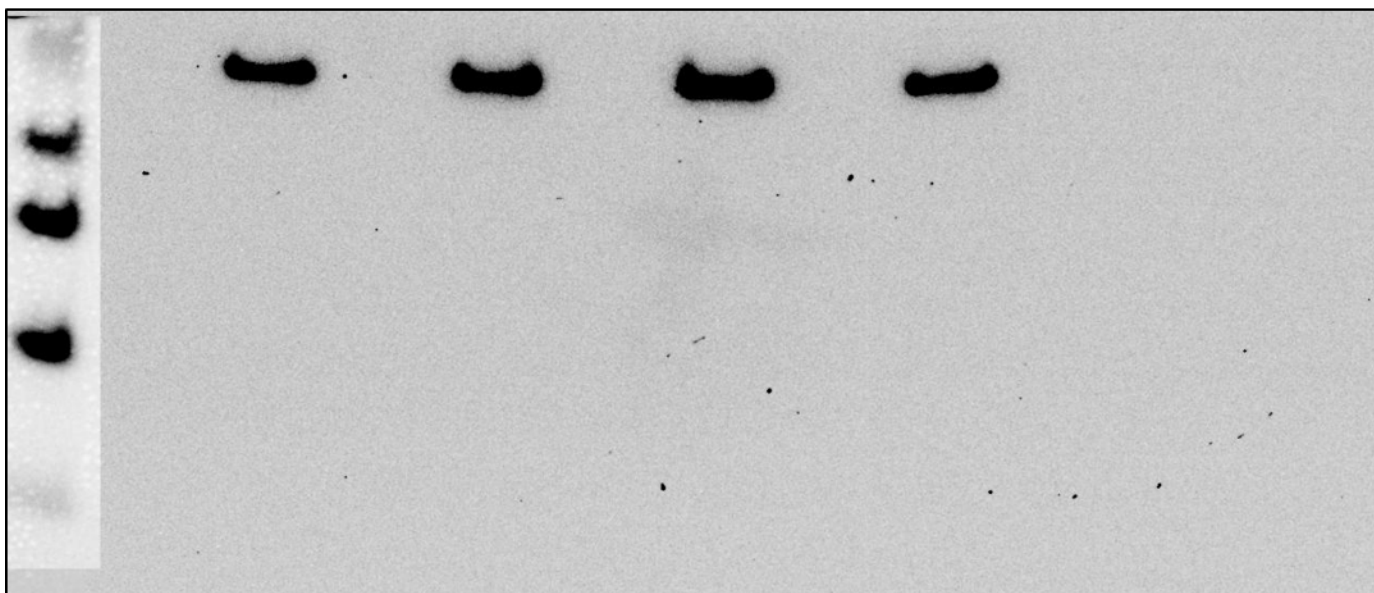

Supplement: Supplementary file 1 [file cancers-15-04328-s001.zip › cancers-2531534-File S1.pdf]
